# Substrates and Loaded Iron Ions Relative Position Influence the Catalytic Characteristics of the Metalloenzymes *Angelica archangelica* Flavone Synthase I and *Camellia sinensis* Flavonol Synthase

**DOI:** 10.3389/fphar.2022.902672

**Published:** 2022-06-08

**Authors:** Zhen Wang, An Liu, Juan Liu, Xu Huang, Feiyao Xiao, Miaomiao Tian, Shenghua Ding, Si Qin, Yang Shan

**Affiliations:** ^1^ Longping Branch Graduate School of Hunan University, Changsha, China; ^2^ Hunan Agricultural Product Processing Institute, Hunan Academy of Agricultural Sciences, Changsha, China; ^3^ Hunan Institute of Nuclear Agricultural Science and Space Breeding, Hunan Academy of Agricultural Sciences, Changsha, China; ^4^ Hunan Province International Joint Laboratory on Fruits and Vegetables Processing, Quality and Safety, Changsha, China; ^5^ College of Food Science and Technology, Hunan Agricultural University, Changsha, China

**Keywords:** synthetic biology, 2-oxoglutarate-dependent dioxygenase, fragment replacement, catalytic characteristics, molecular docking

## Abstract

Metalloenzymes are a class of enzymes that catalyze through the metal ions they load. *Angelica archangelica* flavone synthase I (AnFNS I) and *Camellia sinensis* flavonol synthase (CaFLS), both of which belong to metalloenzymes, have highly similar structures and metal catalytic cores. However, these two enzymes catalyze the same substrate to produce significantly different products. To identify the cause for the differences in the catalytic characteristics of AnFNS I and CaFLS, their protein models were constructed using homology modeling. Structural alignment and molecular docking was also used to elucidate the molecular basis of the differences observed. To analyze and verify the cause for the differences in the catalytic characteristics of AnFNS I and CaFLS, partial fragments of AnFNS I were used to replace the corresponding fragments on CaFLS, and the catalytic characteristics of the mutants were determined by bioconversion assay in *E. coli* and *in vitro* catalytic test. The results suggest that the difference in catalytic characteristics between AnFNS I and CaFLS is caused by the depth of the active pockets and the relative position of the substrate. Mutant 10 which present similar dock result with AnFNS I increased the proportion of diosmetin (a flavone) from 2.54 to 16.68% and decreased the proportion of 4′-O-methyl taxifolin (a flavanol) from 47.28 to 2.88%. It was also indicated that the atoms in the substrate molecule that determine the catalytic outcome may be H-2 and H-3, rather than C-2 and C-3. Moreover, it is speculated that the change in the catalytic characteristics at the changes relative spatial position of H-2/H-3 of hesperetin and the loaded carbonyl iron, caused by charged residues at the entrance of the active pocket, is the key factor for the biosynthesis of flavone from flavanone.

## Introduction

Metalloenzymes are a class of enzymes with loaded metal ions as the catalytic core and play a key role in most metabolic pathways, such as the synthesis of secondary metabolites of plants (Turvey and Grant 1990; Chaplin 2016). Flavones are important secondary metabolites of plants and play an important role in stress resistance (Winkel 2002; Treutter 2006). These metabolites are currently used as the main components of various clinical drugs and functional foods because of their physiological activities including antioxidant (Catarino et al., 2014), anticancer (Lascala et al., 2018), and vasodilation activities (Tettey et al., 2019). However, due to the high consumption of high-purity flavones and the pollution caused by the employed synthetic methods, the biosynthesis of flavones has generated much interest.

**FIGURE 1:**
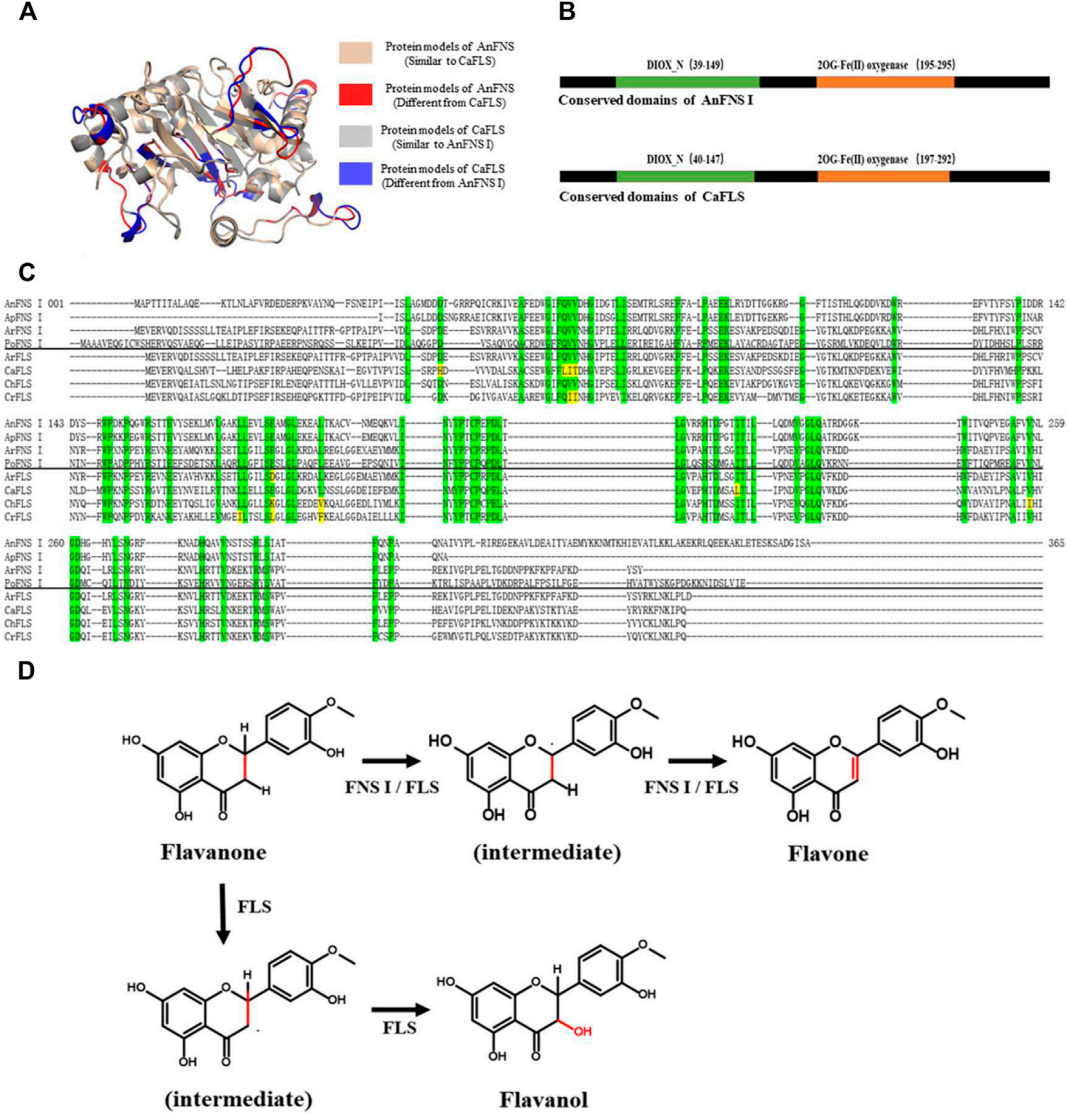
Structure and Catalytic Process of FNS/FLS. Note: **(A)** Structural differences between AnFNS I and CaFLS (The portions of the protein models with significant structural differences are displayed in different colors. Brown and red show the structure of wild-type CaFLS. Gray and blue show the structure of wild-type AnFNS I. Also red and blue indicates the portion of the wild-type CaFLS that differed significantly from AnFNS Ⅰ) **(B)** The conserved domain difference between AnFNS I and CaFLS. **(C)** The conserved amino acid differences between multiple CaFLS and FNS (**AnFNS** I Flavone synthase I of *Angelica archangelica*; **ApFNS** I Flavone synthase I of *Apium graveolens*; **PoFNS** I Flavone synthase I of *Pohlia nutans*;**ArFNS** I Flavone synthase I of *Arabidopsis lyrata*; **ArFLS** Flavonol Synthase of *Arabidopsis thaliana;* **CaFLS** Flavonol Synthase of *Camellia sinensis;* **ChFLS** Flavonol Synthase of *Chrysanthemum x morifolium*; **CrFlS** Flavonol Synthase of *Crocosmia x crocosmiiflora.* Green is annotated with conserved amino acids, yellow with non conserved amino acids) **(D)** Reaction process of synthesis of Flavone from Flavanone catalyzed by FNS/FLS.

The biogenic synthesis of flavones commences with the phenylpropane pathway. In the pathway, Phenylalanine was used to synthesize flavanones (such as naringenin. Harborne and Mabry, 1982) and further to synthesize flavones by hydroxylation and desorption reactions (Turnbull et al., 2004; Cheng et al., 2014). The conversion of flavanone to flavone is catalyzed by FNS I, FNS II, or FLS. The three classes of enzymes belong to metalloenzymes that have the loaded iron ions as their catalytic core. FNS II belongs to the cytochrome P450 family and possesses cytochrome P450 monooxygenase activity which was required to function in conjunction with cytochrome reductases or the microsomal membranes of eukaryotes (Davydov et al., 2015). FNS I and FLS belong to the 2-Oxoglutarate-dependent dioxygenase (2ODGs, Kawai et al., 2014) superfamily (FNS I into DOXC28 and FLS into DOXC47). Both FNS Ⅰ and FLS can exert catalytic activity *in vitro* (Halbwirth et al., 2006; Wang Y. et al., 2021; Wei et al., 2021). The FNS I has a similar structure to the FLS, and the conserved amino acids of FNS I from different sources are also highly conserved in FLS. Therefore, the biosynthesis of flavones by FNS I or FLS may be similar (Figure 1). Despite this, the catalytic characteristics of FNS I and FLS were observed to be significantly different. When hesperetin was used as a substrate for catalysis, diosmetin (a flavone) was the major catalytic product of FNS, and 4′-O-methyl taxifolin (a flavanol) was the major catalytic product of FLS (Wang Z. et al., 2021). Lukačin et al. and Martens et al. suggested that the difference in the catalytic characteristics between FNS Ⅰ and FLS was due to the presence of key amino acid residues in the active pocket (Lukačin and Britsch 1997; Martens et al., 2003), these key amino acid residues may affect the relative spatial position of the substrate with the loaded metal ions. They also indicated that FNS I may have an independent functional region with flavanol C-3 elimination.

Differences in catalytic characteristics can affect the application of this technology to drug synthesis. For structurally highly similar flavonoids, the physiological activity may differ significantly from the effective dose. For example, the structures of diosmetin and hesperetin differ by only one carbon-carbon double bond, and diosmetin is more positive in physiological activity, and diosmetin can achieve the same effect of lower doses for the same physiological function. For example, both diosmetin and hesperetin can inhibit tumor division by binding serine/threonine protein kinase 1 (PLK-1). But the binding ability of diosmetin to PLK-1 (Binding energy of 48.88 kcal/mol) is more potent than that of hesperetin (Binding energy of 46.50 kcal/mol) (Alajmi et al., 2018). Similarly, diosmetin and hesperetin can improve the efficacy or reduce the side effects of anticancer drugs, such as paclitaxel, by inhibiting the activity of CYP2C8. The effect of diosmetin is 16-fold that of hesperetin (mean IC50 of 4.25 and 68.5 µM for diosmetin and hesperetin, respectively) (Quintieri et al., 2011).

Therefore, the reason for the observed differences in the catalytic activity between FNS I and FLS warrants further investigation. In this study, *Angelica archangelica* FNS I (AnFNS I) and *Camellia sinensis* FLS (CaFLS) with similar substrate conversion rate (about 40–50%) were selected as the research objects. Protein models were constructed using homology modeling, and the substrate-binding was modeled with molecular docking. CaFLS was bio-transformed into AnFNS I by fragment substitution to construct mutants, and recombinant *Escherichia coli* displaying heterologous expression of these mutants were constructed. The catalytic activity of these mutants were characterized via bioconversion assay in *E. coli* to verify this hypothesis.

## Materials and Methods

### Genes, Bacterial Strains, Genetic Manipulation, and Chemicals

All genes, bacterial strains, and plasmids used in this study are listed in Table 1. All CaFLS mutants used in this study are listed in Table 2. The gene and protein sequences used in this study are listed in Supplementary materials S1, S2, respectively. All the wild-type genes and mutanted genes were synthesized and sequenced by Sangon Biotech (Shanghai, China). The restriction nuclease was obtained from Sangon Biotech (Shanghai, China). Hesperetin and diosmetin was procured from Macklin (Shanghai, China) and Sigma-Aldrich (St. Louis, MO, United States). Diosmetin, 4′-*O*-methyl taxifolin, and tamarixetin reference material were purchased from Sigma-Aldrich. Isopropyl-β-D-thiogalactoside (IPTG), Luria-Bertani broth (LB), and kanamycin were purchased from Solarbio (Beijing, China).

**TABLE 1:** Genes, plasmids and bacterial strains used.

Gene/Plasmid/Strains	Relevant Characteristics	Source
Gene		
*AnFNS Ⅰ*	Flavone synthase I from *Angelica archangelica,* DQ683352.1	NCBI
*ApFNS Ⅰ*	Flavone synthase I from *Apium graveolens*, KC820132.1	NCBI
*ArFNS Ⅰ*	Flavone synthase I from *Arabidopsis lyrata*, AM887659.1	NCBI
*PoFNS Ⅰ*	Flavone synthase I from *Pohlia nutans*, MK036763.1	NCBI
*ArFLS*	Flavonol synthase from *Arabidopsislyrata*, NM_120,951.3	NCBI
*CaFLS*	Flavonol synthase from *Camellia sinensis*, MH232962.1	NCBI
*ChFLS*	Flavonol synthase from *Chrysanthemumxmorifolium*, MF124607.1	NCBI
*CrFLS*	Flavonol synthase from *Crocosmia x crocosmiiflora*, MK562524.1	NCBI
*Mutant 01*	Flavonol synthase mutant constructed using CaFLS as template	This study
*Mutant 02*	Flavonol synthase mutant constructed using CaFLS as template	This study
*Mutant 03*	Flavonol synthase mutant constructed using CaFLS as template	This study
*Mutant 04*	Flavonol synthase mutant constructed using CaFLS as template	This study
*Mutant 05*	Flavonol synthase mutant constructed using CaFLS as template	This study
*Mutant 06*	Flavonol synthase mutant constructed using CaFLS as template	This study
*Mutant 07*	Flavonol synthase mutant constructed using CaFLS as template	This study
*Mutant 08*	Flavonol synthase mutant constructed using CaFLS as template	This study
*Mutant 09*	Flavonol synthase mutant constructed using CaFLS as template	This study
*Mutant 10*	Flavonol synthase mutant constructed using CaFLS as template	This study
Plasmid		
pET-28a (+)	T7 promoter, LacO, Kanamycin resistant gene	Novagen
Strains		
*E. coli* TOP10	F^−^ *mcrA* Δ(mrr^-^hsdRMS^-^mcrBC) *φ80lacZ*Δ*M15* Δ*lacX74 nupG recA1 araD139* Δ(ara^-^leu)7697 *galE15 galK16 rpsL* (StrR) *endA1 λ* ^*-*^	Sangon Biotech
*E. coli* BL21 (DE3)	F^−^ *ompT hsdSB*(r_B_^−^m_B_^−^) *gal dcm lon* (DE3)	Novagen

**TABLE 2:** Mutants by fragment replacement.

Mutants	Replaced fragments of CaFLS, AA	Corresponding fragments of AnFNS Ⅰ, AA	Relevant Characteristics
Mutant 01	100–113	105–116	Adjust the depth of the active pocket
Mutant 02	236–241	237–244	Increase the active pocket commonly found in FNS Ⅰ but not in CaFLS
Mutant 03	322–330	323–364	Extend C-terminal α-helix
Mutant 04	211–216	212–217	The amino acids near iron ion binding sites (217) and differentially charged residues (215) were replaced
Mutant 05	270–276	273–279	The amino acids near iron ion binding sites (273) were replaced
Mutant 06	211–216, 270–276	212–217, 273–279	The amino acids near multiple iron ion binding sites were replaced
Mutant 07	220–223	221–224	The amino acids near the hesperetin binding site which close to iron ion binding site were replaced
Mutant 08	188–198	191–199	The amino acids near the hesperetin binding site on β-sheet 5 (β5) and differentially charged residues (198) were replaced
Mutant 09	285–293	288–296	The amino acids near the hesperetin binding site on β-sheet 11 (β11) were replaced
Mutant 10	126–131	129–134	The amino acids near the hesperetin binding site on β-sheet 3 (β3) and differentially charged residues (126) were replaced

### Homology Modeling, Active Pocket, and Substrate Binding Site Prediction

The homology modeling website SWISS-MODEL (https://swissmodel.expasy.org) was used to construct a three-dimensional model of these proteins which used in the study, and the online software SAVES 6.0 (https://saves.mbi.ucla.edu) was used to evaluate the quality of the models. The molecular structures of hesperetin, 4′-*O*-methyl taxifolin, and diosmetin were prepared and optimized using Indraw 5.2. (Shanghai Yinggu Information Technology Co., Ltd. Shanghai, China). The molecular structure of the iron ligand (carbonyl iron) was fabricated and optimized using Indraw 5.2, according to Welford et al. (2005). The active pockets of the proteins were then analyzed using POCASA 1.1 (http://g6altair.sci.hokudai.ac.jp/g6/service/pocasa/). The radius (Å) of the probe sphere was 2.0 Å and the single-point flag was 16. The single-point flag used was 18, and the grid size was 1.0 Å. The substrate-binding sites of the proteins were determined using COACH (https://zhanglab.ccmb.med.umich.edu/COACH/) which was provided by (Yang et al., 2013a; Yang et al., 2013b).

### Sequence Alignment, Model Alignment, and Molecular Docking

Sequence alignment was performed using the multiple sequence alignment tool (https://www.novopro.cntools/muscle.html, Edgar 2004) provided by Neoprobio Inc. (Weihai, China). Protein model alignment was performed using PyMol 1.7 software (Schrödinger, Inc. New York, NY, United States), and molecular docking was performed using AutoDock Vina 1.1.2 software (Olson Laboratory, Scripps Research, La Jolla, CA, United States) according to Trott and Olson, (2010). The selection of the preferred conformation for molecular docking was based on the following principle:

**Proteins vs iron ligands**: The iron ligands are conformed to a predicted active binding site, with lower binding energy.

**Proteins vs hesperetin**: hesperetin is located within the active pocket; hesperetin does not sterically overlap with the iron ligands.

### Construction of Recombinant *E. coli*

The synthesized mutant genes were connected into a pET-28a (+) plasmid via NcoI/XhoI double digestion, and the plasmid was transformed into *E. coli* TOP10 for amplification. The plasmid was then extracted and transferred into *E. coli* BL21 (DE3) cells. The pET-28a (+) without any additional genes was also transferred to independent *E. coli* BL21 (DE3) as negative control. The screening for positive bacteria was performed using LB solid medium containing kanamycin (100 mg/L).

### Bioconversion Assay in *E. coli* and Catalytic Characteristics Analysis

Recombinant *E. coli* was cultured in LB liquid medium at 37°C to an OD_600_ of 0.6–0.8. Protein expression was then induced for 12 h at 18°C after adding isopropyl-β-D-thiogalactoside at a final concentration of 0.3 mM. Subsequently, at a final concentration of 100 mg/L, hesperetin was added as the reaction substrate and was left to react for 24 h at 18°C. Protein samples were separated by SDS-PAGE. The gel was dyed and decolorized using the Coomassie brilliant blue method. The approximate molecular weight of the protein was determined by comparison with 15–130 kDa protein markers.

Bioconversion assay in *E. coli*: Recombinant *E. col*i strains were induced to express FNS/FLS as described above. Hesperetin (final concentration 100 mg/L), α-ketoglutaric acid (final concentration 100 mg/L), and ferrous sulfate (final concentration 1 g/L) were added to the reconstituted *E. coli* broth as the reaction substrate. The recombinant *E. coli* broth containing the reaction substrate was cultured at 23°C for 24 h to synthesize diosmetin.

*In vitro* catalysis: The *in vitro* catalytic medium described by Stotz et al. 1981) was used at 2 ml total volume containing 0.1 mM Tris-HCl (pH 7.5), 5 mM 2-mercaptoethanol, 0.3 mM hesperetin, 1 mM NADPH, and 0.1 mg separated protein. The reaction was conducted at 23°C for 1 h to verify the catalytic activity of FNS/FLS *in vitro*. EDTA, ascorbic acid, ferrous sulfate, copper chloride, magnesium chloride, zinc chloride, and calcium chloride were added as cofactors to determine the effect on catalytic activity. Km and Vmax were determined by the Lineweaver-Burk method.

The detection method of the products was referred to Wang Z. et al. (2021) Flavonoids were characterized using liquid chromatography–mass spectrometry and nuclear magnetic resonance (NMR). Hesperetin, diosmetin, 4′-*O*-methyl taxifolin, and tamarixetin were then quantified using ultra-performance liquid chromatography (UPLC) with the Agilent 1290 UPLC instrument and Agilent Q-TOF 6550 MS system (Agilent Technologies, Santa Clara, CA, United States). The UPLC apparatus was equipped with an Agilent Extend C18 column (2.1 × 50 mm, 1.7 μm), and the mobile phase was 10% acetonitrile in water. The mass spectrum scanning range was 50–400 m/z, gas temperature was 150°C, drying gas rate was 15 L/min, gas temperature was 350°C, shear gas flow rate was 12 L/min, electrospray ionization mode voltage was 4000 V, and fragment voltage was 175 V. Prior to NMR analysis, the synthesized diosmetin was used to prepare the liquid phase (LC-20AP, Shimadzu, Kyoto, Japan). The samples were lyophilized and dissolved in deuterated methanol. The ^1^HNMR (600.22 MHz) analysis was performed on the Bruker Avance-600 spectrometer (Billerica, MA, United States), using a pulse sequence of RD 90°–t1–90°–tm–90° to acquire the free induction decay. The Acquity UPLC system was equipped with a BEH C18 column (2.1 × 50 mm, 1.7 μm; Waters, Milford, MA, United States). Water and methanol were used as the mobile phases. The detection wavelength was set at 330 nm. All the results were controlled using standard reference materials.

The substrate conversion is calculated by Eq. 1:

$$\alpha_{1}=\left(1-\frac{n_{1}}{n_{0}}\right)\times 100\%$$ (1)

In Eq. 1:

α_1_: Substrate conversion, %.

*n*_0_: Total amount of added substrate, mM.

*n*_*1*_: Total amount of substrate in the system at the end of bioconversion, mM.

The products conversion is calculated by Eq. 2:

$$\alpha_{2}=\frac{n_{2}}{n_{0}}\times 100\%$$ (2)

In Eq. 2:

α_2_: Products conversion, %.

*n*_0_: Total amount of added substrate, mM.

*n*_*2*_: Total amount of products in the system at the end of bioconversion, mM.

### Statistical Analysis

The statistical analysis of the results was performed using the SPSS version 25.0 software (SPSS, Inc. Chicago, IL, United States), and the analysis method is least—significantdifference. Plotted using the GraphPad Prism eight software (GraphPad, Inc. San Diego, CA, United States).

## Results

### Catalytic Characteristics and Structural Differences Between AnFNS I and CaFLS

**FIGURE 2:**
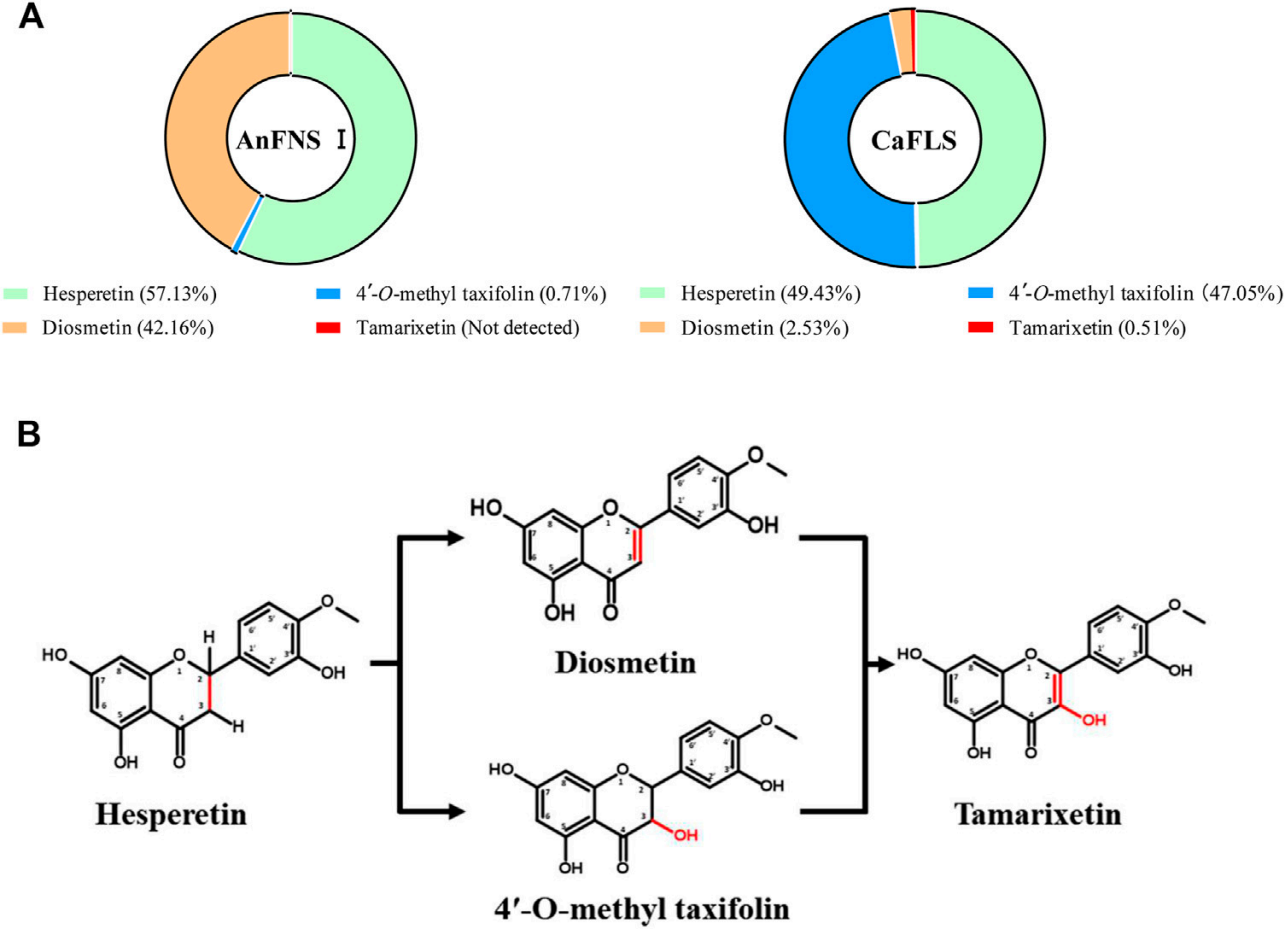
Catalytic products of hesperetin catalyzed by AnFNS I and CaFLS. Note: **(A)** Proportion of multiple flavonoids in the catalytic products. **(B)** Possible formation processes of multiple flavonoids (**Not detected:** Refers to concentrations below the detection limit).

**FIGURE 3:**
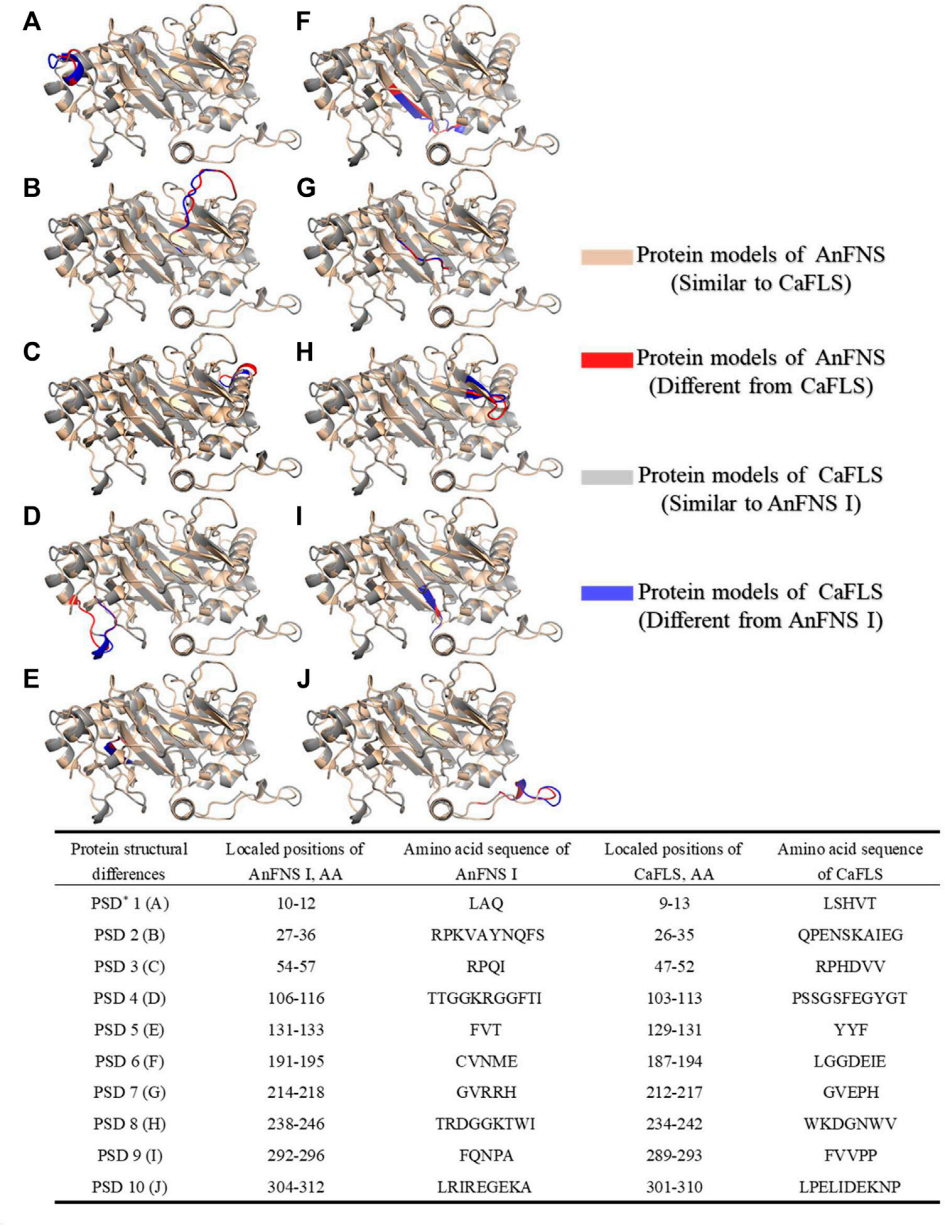
Major structural differences between AnFNS I and CaFLS. Note: **(A)** Protein structural differences 1 (PSD 1). **(B)** Protein structural differences 2 (PSD 2). **(C)** Protein structural differences 3 (PSD 3). **(D)** Protein structural differences 4 (PSD 4). **(E)** Protein structural differences 5 (PSD 5). **(F)** Protein structural differences 6 (PSD 6). **(G)** Protein structural differences 7 (PSD 7). **(H)** Protein structural differences 8 (PSD 8). **(I)** Protein structural differences 9 (PSD 9). **(J)** Protein structural differences 10 (PSD 10) (The portions of the protein models with significant structural differences are displayed in different colors. Brown and red show the structure of wild-type CaFLS. Gray and blue show the structure of wild-type AnFNS I. Also red and blue indicates the portion of the wild-type CaFLS that differed significantly from AnFNS I).

**FIGURE 4:**
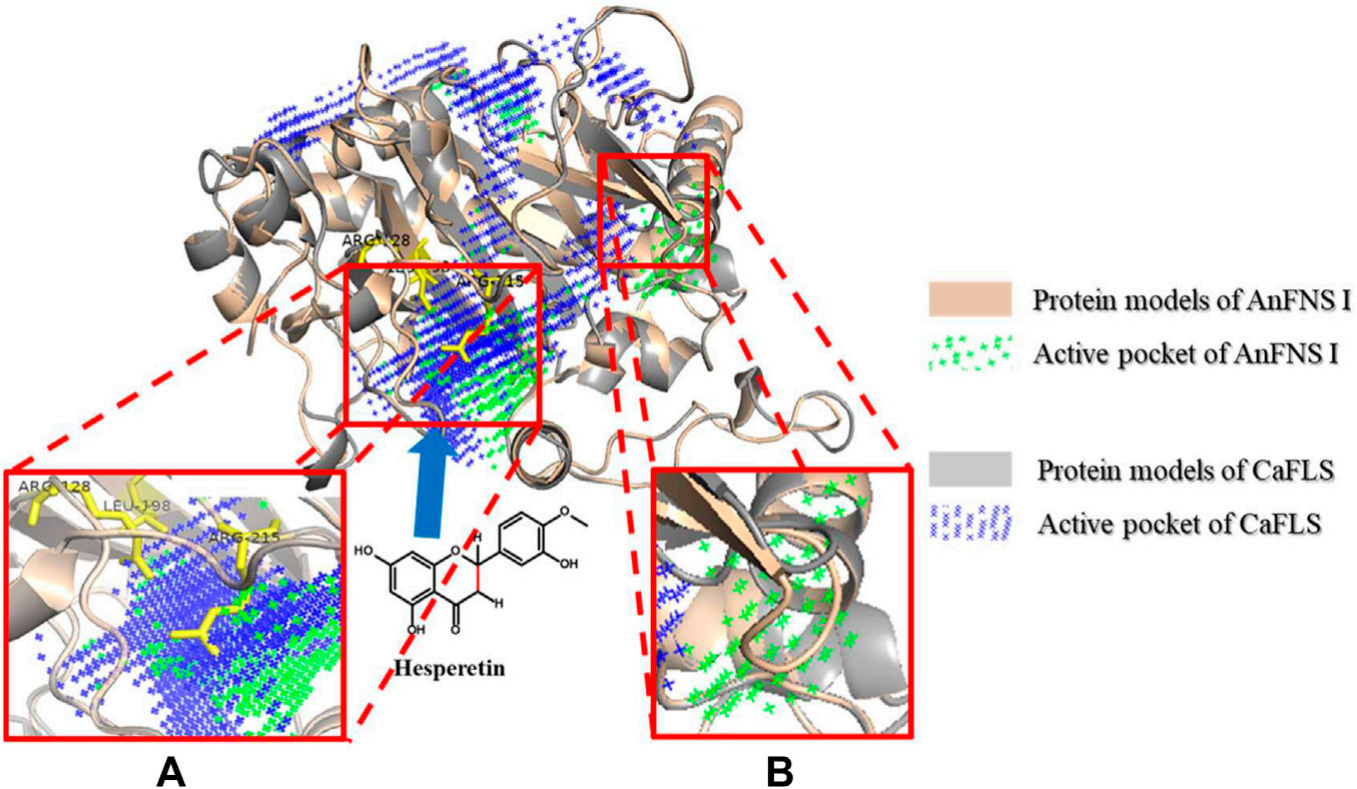
Major active pocket difference between AnFNS I and CaFLS. Note: **(A)** Active pocket difference 1 (APD 1). **(B)** Active pocket difference 2 (APD 2). Blue arrows indicate the direction of substrate movement into APD 1 by the entrance.

The products obtained from the catalysis of hesperetin by AnFNS I and CaFLS are shown in Figure 2. The substance qualitative results are presented in Supplementary material S3. Diosmetin, 4′-*O*-methyl taxifolin, and tamarixetin were not detected in the broth of recombinant *E. coli* from the negative group which not carrying any FLS or FNS Ⅰ genes. The catalytic products of AnFNS I was dominated by diosmetin, and the catalytic products of CaFLS contained diosmetin, 4′-*O*-methyl taxifolin, and tamarixetin, with 4′-*O*-methyl taxifolin accounting for most of the products. Information on the homology modeling of AnFNS I with CaFLS is given in Table 3. Although the protein models of AnFNS I and CaFLS were truncated at the N- and C-termini compared to the actual sequence, we considered the model to be a correct representation of the approximate structure of the protein with the active center. This was confirmed as the model versus template sequence identity both exceeded 0.3, the coverage was above 89%, and high scores were obtained in the SAVES analysis. The sequence identity of AnFNS I with CaFLS was 31.4%, and the overall RMSD was less than 0.4 Å (0.37 Å, using the SSM algorithm in Coot), indicating that they were less structurally dissimilar. The Distance Constraint (Studer et al., 2019) and Overall Quality Factor (Colovos and Yeates 1993) of the protein models show that the model are reliable. A sequence alignment of the FNS I and FLS from eight origins was performed (Figure 1C), and a total of 64 conserved amino acids were found in the FNS Ⅰ from different origins. These amino acids are also mostly conserved in FLS of different origins. ArFLS was the most highly conserved (98.4%) and CaFLS was the least conserved (91.2%). Alignment of the structural differences between AnFNS I and CaFLS revealed 10 significant differences designated as protein structural differences (PSD) 1–10, as shown in Figure 3; Table 4. Among the PSD, PSD 1 and PSD 2 displayed to the N-terminal structural differences. PSD 3–5 were observed in the DIOX_N conserved domain, whereas PSD 6 was identified between the DIOX_N and 2OG-Fe(II) oxygenase conserved domains. PSD 7–10 were observed in the 2OG-Fe(II) oxygenase conserved domain. Notably, part of these differences were identified on the surface of the protein, and far from the protein active site or active pocket. In addition, the C-terminal helix length of AnFNS I was remarkable longer than that of CaFLS by 32 amino acids. However, the model obtained from homology modeling only extended to 327 AA of the protein (total amino acids of AnFNS I were 365 AA, and 331 AA for CaFLS), which was not fully represented in the model. The active pocket difference (APD) of AnFNS I with CaFLS, 1 and 2, are shown in Figure 4.

**TABLE 3:** Homology modeling information of AnFNS I, CaFLS, and Mutants.

Proteins	Template	Seq Identity (%)	Coverage	Range, AA	Distance Constraint	Overall Quality Factor
AnFNS I	6lsv.1.A^a^	32.41	0.89	5–330	0.69 ± 0.05	92.3077
CaFLS	1gp4.1 A^b^	45.26	0.99	3–329	0.79 ± 0.05	92.2830
Mutant 1	1gp4.1.A	44.17	0.99	2–327	0.78 ± 0.05	93.5484
Mutant 2	1gp4.1.A	44.95	0.98	3–331	0.78 ± 0.05	90.4459
Mutant 3	1gp4.1.A	44.82	0.90	2–329	0.78 ± 0.05	95.8333
Mutant 4	1gp4.1.A	44.51	0.99	2–329	0.79 ± 0.05	91.9872
Mutant 5	1gp4.1.A	43.90	0.99	2–329	0.78 ± 0.05	94.2308
Mutant 6	1gp4.1.A	43.29	0.99	2–329	0.78 ± 0.05	95.8333
Mutant 7	1gp4.1.A	43.87	0.99	2–327	0.78 ± 0.05	95.1923
Mutant 8	1gp4.1.A	43.87	0.99	2–327	0.78 ± 0.05	91.2903
Mutant 9	1gp4.1.A	44.21	0.99	2–329	0.78 ± 0.05	93.2692
Mutant 10	1gp4.1.A	44.04	0.99	3–329	0.79 ± 0.05	89.0675

a 6lsv.1.A means Crystal structure of jasmonate-induced oxygenase 2 (JOX2) in complex with 2OG, fe, and JA. The best hits of AnFNS I.

b 1gp4.1.A means Anthocyanidin synthase from *Arabidopsis thaliana*. The best hits of wild-type CaFLS, and Mutants.

**TABLE 4:** Protein structural differences between AnFNS I and CaFLS.

Protein structural differences	Localed positions of AnFNS I, AA	Amino acid sequence of AnFNS I	Localed positions of CaFLS, AA	Amino acid sequence of CaFLS
PSD^a^ 1 (A)	10–12	LAQ	9–13	LSHVT
PSD 2 (B)	27–36	RPKVAYNQFS	26–35	QPENSKAIEG
PSD 3 (C)	54′-57	RPQI	47–52	RPHDVV
PSD 4 (D)	106–116	TTGGKRGGFTI	103–113	PSSGSFEGYGT
PSD 5 (E)	131–133	FVT	129–131	YYF
PSD 6 (F)	191–195	CVNME	187–194	LGGDEIE
PSD 7 (G)	214′-218	GVRRH	212–217	GVEPH
PSD 8 (H)	238–246	TRDGGKTWI	234′-242	WKDGNWV
PSD 9 (I)	292–296	FQNPA	289–293	FVVPP
PSD 10 (J)	304′-312	LRIREGEKA	301–310	LPELIDEKNP

a PSD, means protein structural differences.

**FIGURE 5:**
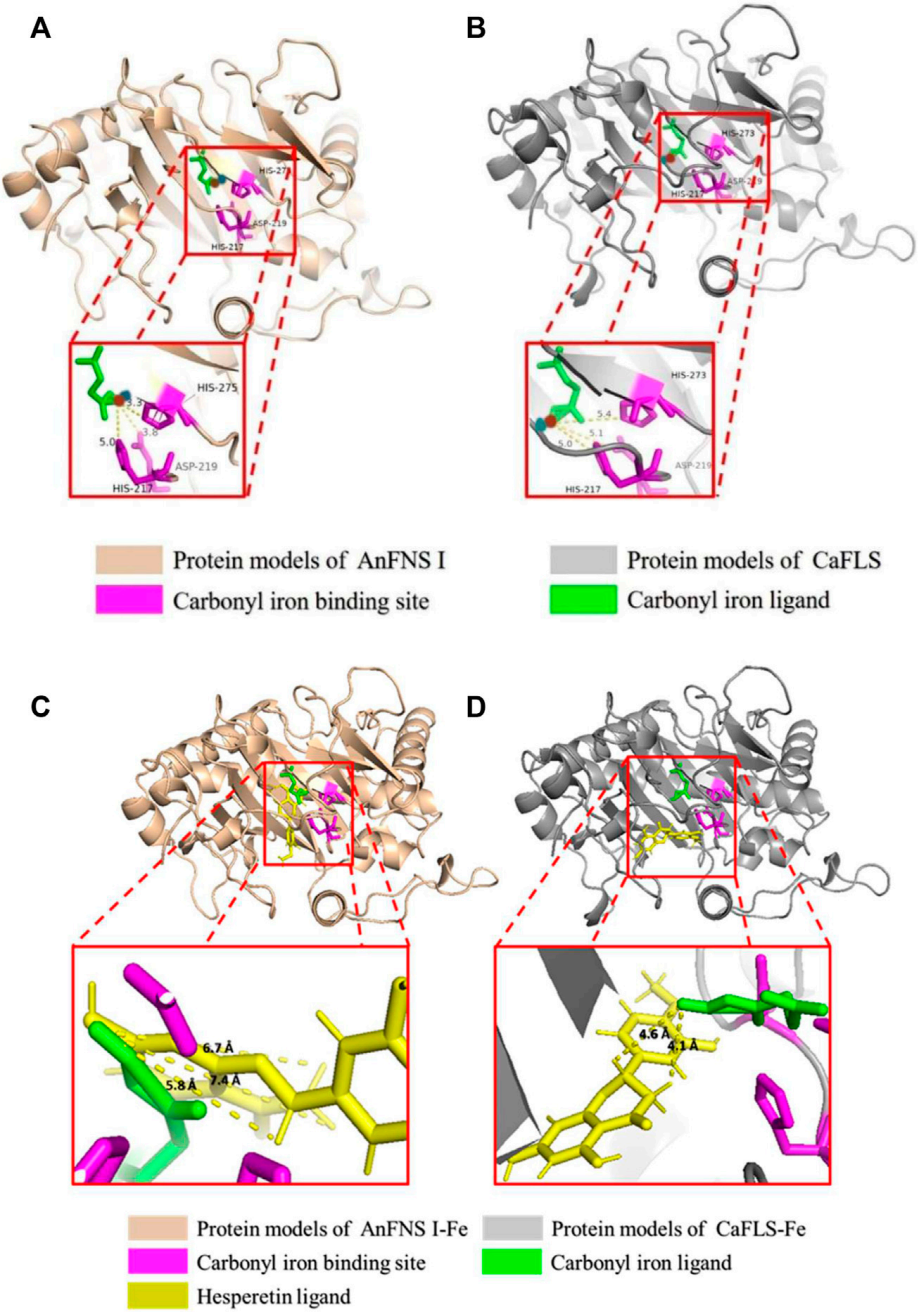
Binding conformation of AnFNS I/CaFLS with Carbonyl iron and Hesperetin. Note: **(A)** Binding conformation of AnFNS I with Carbonyl iron. **(B)** Binding conformation of CaFLS with Carbonyl iron (Blue dots represent oxygen atoms and red dots represent iron atoms). **(C)** Binding conformation of AnFNS I with Hesperetin. **(D)** Binding conformation of CaFLS with Hesperetin.

The binding of AnFNS I and CaFLS to iron ligands is shown in Figures 5A,B. The iron ion binding sites of AnFNS I were identified as H217, D219, and H275, and the iron ion is loaded on the bottom of the active pocket as catalytic core. CaFLS displayed a similar binding site of H217, D219, and H273. As such, there is a difference in the orientation of the carbonyl in the iron ligand, which is closer to the active pocket center in AnFNS I. For CaFLS this orientation is further away from the active pocket center. The protein was docked with hesperetind, and the binding conditions are shown in Figures 5C,D. Although hesperetin was identified in APD 1, when bound to both AnFNS I and CaFLS, the two spatial locations differed significantly. The A and C rings of hesperetin were closer to the bottom of APD 1 in AnFNS I, whereas for CaFLS, they were located near the top of APD 1. The docking results of AnFNS I with hesperetin revealed that the A ring of hesperetin is located between the carbonyl of the iron ligand and H-3, which may cause AnFNS I to lose C-3 catalytic activity and unable to hydroxylate C-3. Although some of the docking conformations have displayed a lower binding energy than the selected conformation, these conformations are considered unreliable due to the spatial superposition of the iron ligand and hesperetin.

Therefore, 10 mutants were designed for the next experiments featuring fragment replacement of the wild-type CaFLS based on the differences in the active pocket, differences in the charged residues at the entrance of the pocket, and structural differences at the substrate-binding site.

### Catalytic Characteristics of Mutants

SDS-PAGE was used to separate the intracellular soluble proteins expressed from the recombinant *E. coli*, and the result is shown in Supplementary material S4. Expression of wild-type and all mutanted CaFLS was normally induced by IPTG.

The hesperetin bioconversion assay in *E. coli* which heterologously expressed mutated CaFLS. The resultant flavonoids and catalytic characteristics of each mutants are shown in Table 5. Almost all mutants except mutants 01 and 09 showed a significant decrease in catalytic activity toward hesperetin. The mutants 02, 07, and 09 products were similar to wild-type CaFLS, with the catalytic product dominated by 4′-O-methyl taxifolin and contained small amounts of diosmetin. Mutants 03 and 05 almost lost their catalytic ability toward hesperetin. Mutant 08 lost the catalytic activity associated with the biosynthesis of diosmetin. The proportion of tamarixetin in the products of mutant 01 significantly increased. Similarly, the proportion of diosmetin in mutant 10 was also significantly increased, and the catalytic characteristics were similar to those of AnFNS Ⅰ. It was also observed that the proportions of diosmetin and tamarixetin in the products from mutant 04 had increased.

**TABLE 5:** Products conversion rate in bioconversion assay and catalytic activity *in vitro*.

Proteins	Products	Conversion Rate (%)	K_m_ (uM)	V_max_ (nKat·mg^−1^)	K_cat_ ((s^−1^)	K_cat_/K_m_ (s^−1^·mM^−1^)
AnFNS Ⅰ	Diosmetin	42.16	59.04	0.61	0.0249	0.4230
	4′-*O*-methyl taxifolin	0.71	92.58	0.01	0.0004	0.0045
	Tamarixetin	ND	—	—	—	—
CaFLS	Diosmetin	2.53	80.36	0.05	0.0019	0.0236
	4′-*O*-methyl taxifolin	47.05	55.47	0.93	0.0349	0.6286
	Tamarixetin	0.51	96.04	0.01	0.0004	0.0039
Mutant 01	Diosmetin	2.46	79.59	0.05	0.0019	0.0239
	4′-*O*-methyl taxifolin	22.45	67.58	0.46	0.0171	0.2532
	Tamarixetin	24.92	66.41	0.51	0.0190	0.2860
Mutant 02	Diosmetin	1.23	90.44	0.03	0.0015	0.0166
	4′-*O*-methyl taxifolin	40.29	60.66	0.98	0.0369	0.6075
	Tamarixetin	ND	—	—	—	—
Mutant 03	Diosmetin	0.34	98.56	0.01	0.0011	0.0112
	4′-*O*-methyl taxifolin	6.40	85.97	0.19	0.0071	0.0821
	Tamarixetin	ND	—	—	—	—
Mutant 04	Diosmetin	10.62	74.12	0.16	0.0045	0.0607
	4′-*O*-methyl taxifolin	12.03	71.77	0.18	0.0068	0.0947
	Tamarixetin	14.08	68.47	0.21	0.0080	0.1162
Mutant 05	Diosmetin	ND	—	—	—	—
	4′-*O*-methyl taxifolin	1.36	88.59	0.03	0.0011	0.0127
	Tamarixetin	ND	—	—	—	—
Mutant 06	Diosmetin	1.27	89.67	0.02	0.0015	0.0167
	4′-*O*-methyl taxifolin	20.89	64.41	0.33	0.0123	0.1915
	Tamarixetin	1.04	91.88	0.02	0.0006	0.0067
Mutant 07	Diosmetin	1.11	91.57	0.02	0.0015	0.0164
	4′-*O*-methyl taxifolin	31.30	63.54	0.56	0.0211	0.3328
	Tamarixetin	ND	—	—	—	—
Mutant 08	Diosmetin	ND	—	—	—	—
	4′-*O*-methyl taxifolin	24.95	66.53	0.37	0.0090	0.1353
	Tamarixetin	ND	—	—	—	—
Mutant 09	Diosmetin	0.53	89.24	0.01	0.0011	0.0123
	4′-*O*-methyl taxifolin	49.47	54.90	0.93	0.0350	0.6376
	Tamarixetin	ND	—	—	—	—
Mutant 10	Diosmetin	16.68	65.43	0.29	0.0109	0.1660
	4′-*O*-methyl taxifolin	2.88	78.06	0.05	0.0019	0.0243
	Tamarixetin	0.67	88.22	0.01	0.0004	0.0049

ND, means that the substance was not detected.

The results suggested that the active pocket depth, iron ligand, and hesperetin-binding sites of the mutants affected the catalytic characteristics of the enzyme. In contrast, the new active pocket unique to AnFNS I, did not change the catalytic characteristics. Therefore, it was speculated that the difference in catalytic characteristics between AnFNS I and CaFLS may be due to the relative positions of the iron ligands and hesperetin in the active pocket and no other factors.

### Structural Comparison Between Mutants and Wild-Type

**FIGURE 6:**
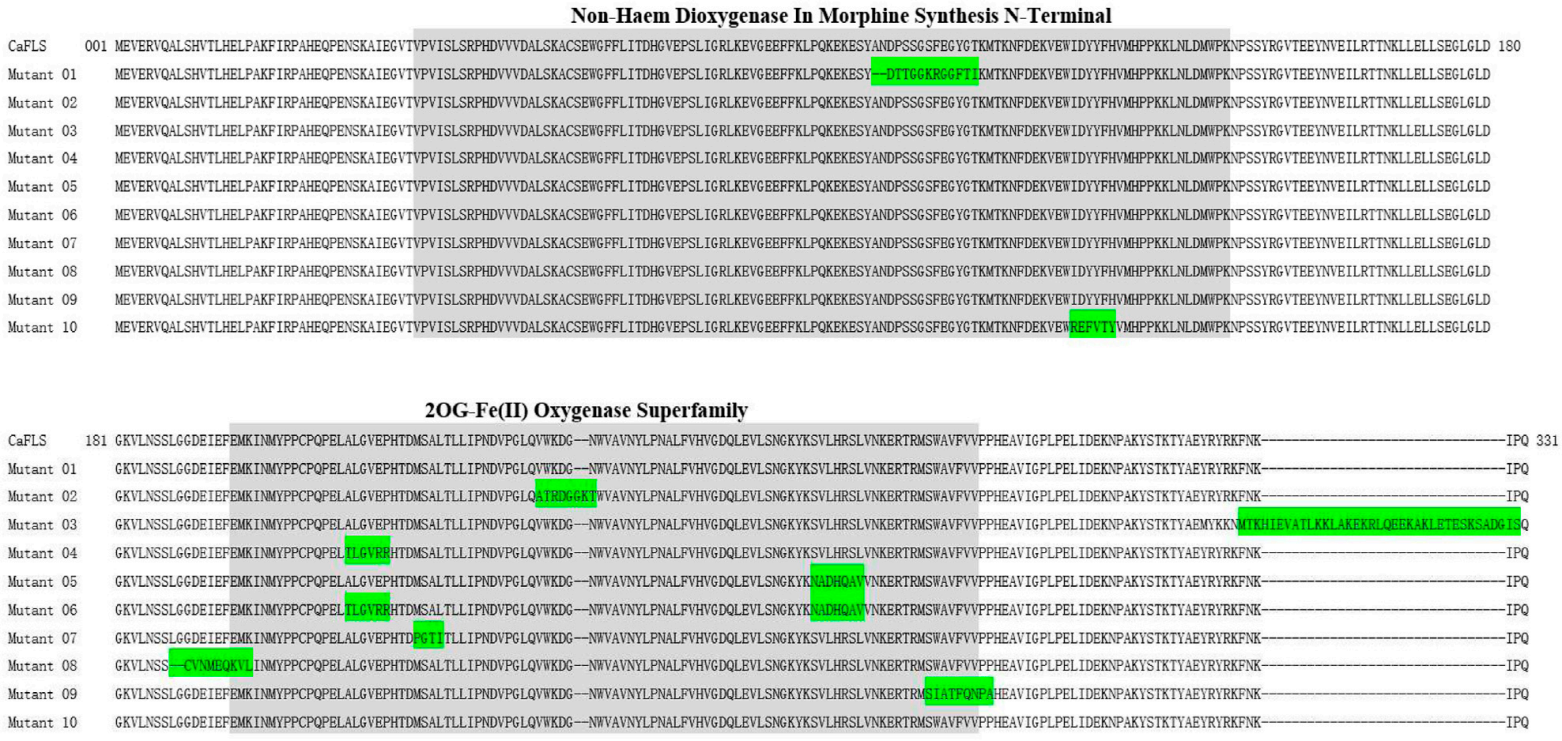
Sequence alignment of mutant CaFLS with wild-type CaFLS. Note: Green indicates divergent sequences, and gray indicates the 2ODGs conserved domain.

**FIGURE 7:**
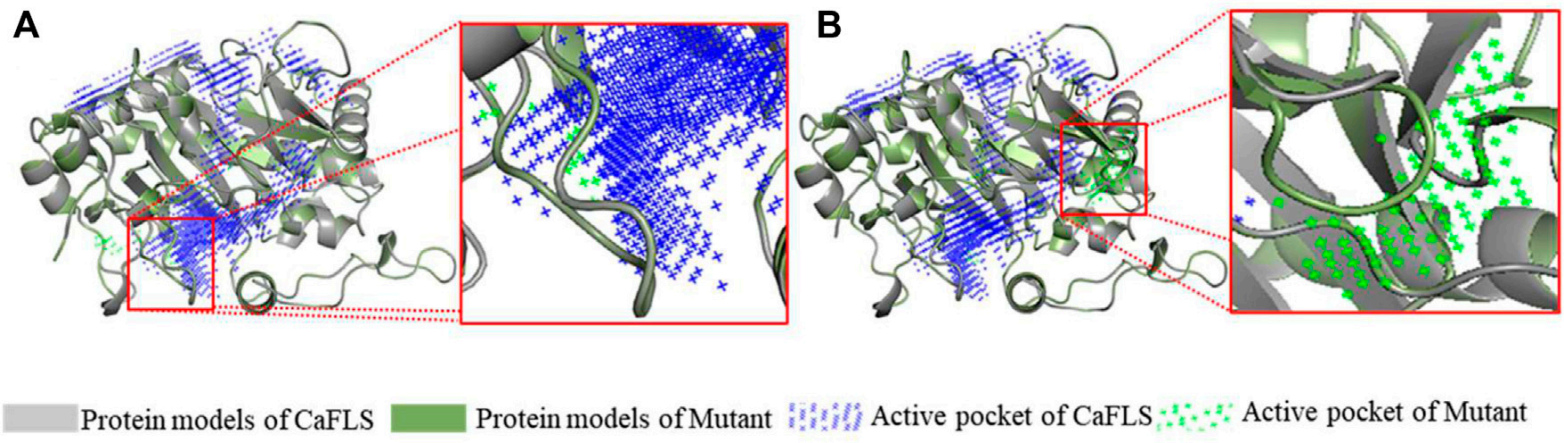
Active pocket differences of mutant and wild-type CaFLS. Note: **(A)** Active pocket differences of mutant 01 and wild-type CaFLS (Alterations in depth). **(B)** Active pocket differences of mutant 02 and wild-type CaFLS (Increases the active pocket).

The mutants were also subjected to homologous modelling using the same approach described previously (Table 3). Sequence alignment of wild-type CaFLS with mutants is shown in Figure 6. The structural differences between mutants 01, 02, and 03 and wild-type CaFLS were compared. The active pockets of mutant 01 displayed shallower depths. In mutant 02, the APD 2 active pocket was similar to that of AnFNS I, as shown in Figure 7. The change in mutant 03 was not represented in the derived model.

### Docking of Mutants With Substrates

**FIGURE 8:**
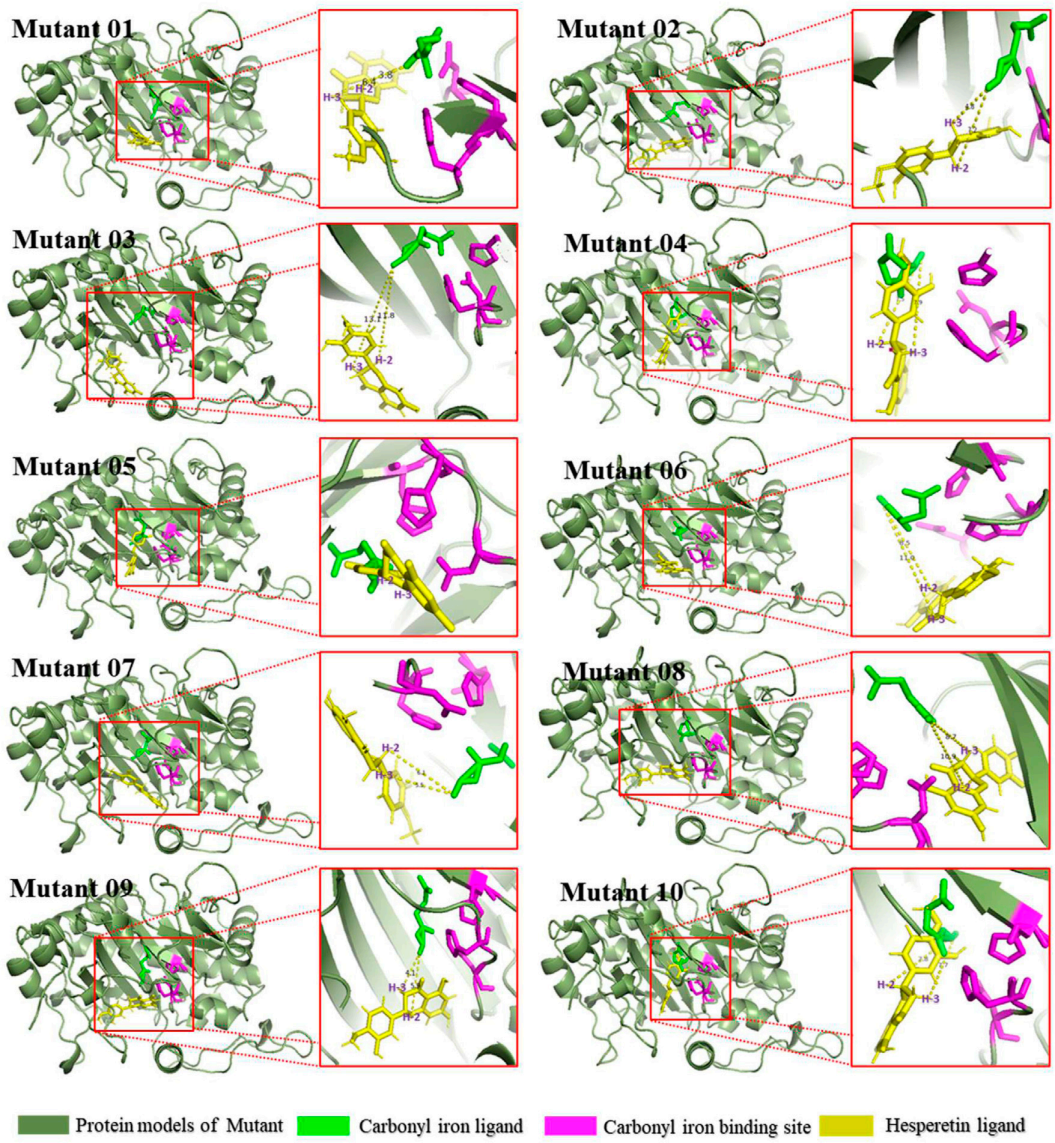
Binding conformations of substrates and mutants.

The mutants were docked independently with iron ligand and hesperidin, and the selected conformation is shown in Figure 8. In mutants 04 and 06, the positions of the iron ligands in the active pocket changed significantly, achieving the design goal. However, in mutant 05, the position of the iron ligand in the active pocket did not change significantly. This may be due to the inferior iron binding ability of H273 compared to that of H217 and D219. Hesperetin was then docked with the mutants, the conformation was selected, and the distance between the carbonyl of the iron ligand and hesperetin H-2 and H-3 was measured. The results are presented in Figure 8. The carbonyls in mutants 01, 04, and 10, were closer to H-2 than H-3. In addition, the proportion of diosmetin or tamarixetin in the catalytic products was increased, indicating that the catalytic capacity of H-2 was stronger than that of wild-type CaFLS. Among them, mutant 10 displayed similar characteristics as AnFNS I. The position of the B ring of hesperetin was located between the carbonyl of the iron ligand and H-3, which affected the catalytic activity of mutant 10 on C-3. This led to the reduction of the ratio between 4′-*O*-methyl taxifolin and tamarixetin in the catalytic products. In mutants 02, 07, 08, and 09, the carbonyl was closer to H-3, and the ratio of diosmetin and tamarixetin did not increase significantly. The catalytic characteristics were similar to those of wild-type CaFLS. Among them, H-2 and the carbonyl moiety in the selected conformation of mutant 08 were located on different sides of the C ring of hesperetin. This resulted in the catalytic products of mutant 08 not being detected. In the selected conformation of mutant 03 and 06, the distance between the carbonyls of the iron ligand and H-2/H-3 of hesperetin was further than that of wild-type CaFLS (which was approximately 4.5 Å in CaFLS and greater than 11 Å in mutant 03). This resulted in a significant decrease in the overall catalytic activity of these mutants. A suitable conformation of mutant 05 with the substrate could not be selected. In addition, hesperetin could not be correctly located in the active pocket and often overlapped with the iron ligand in space. The results of the catalytic experiments showed that the catalytic ability associated with hesperetin was almost lost, and only minimal quantities of hesperetin could be converted to 4′-*O*-methyl taxifolin.

## Discussion

### Catalytic Products of FNS I and CaFLS

Previous studies have found that AnFNS I and CaFLS are highly similar in structure (Wang Z. et al., 2021). To investigate the reason for the differences in the catalytic characteristics between AnFNS I and CaFLS, 10 CaFLS mutants were constructed based on the structural differences between the enzymes. The results revealed that mutants with different replacement strategies showed diverse catalytic characteristics. Some of the mutants catalyzed the transformation of hesperetin to 4′-*O*-methyl taxifolin and diosmetin, and in some instances, tamarixetin (flavonol, which can be regarded as a further catalytic product of diosmetin). Flavanone is catalyzed by FLS to produce a variety of products at the same time, which is consistent with Turnbull et al. reported (Turnbull et al., 2004).

### Influence of the Fragment Replacement and C-Terminal Helix

Martens et al. performed fragment substitution for flavanone 3*β*-hydroxylase (F3T, of 2OGDs) and FNS I. While the N-terminal 219 amino acids of FNS were fused with the C-terminal 146 amino acids of FHT, the fusion protein activity was close to that of FNS. While the N-terminal 219 amino acids of FHT were fused with the C-terminal 149 amino acids of FNS, the fusion protein lost FNS activity (Martens et al., 2001; Martens et al., 2003). The result suggests that the catalytic characteristics of 2OGDs have a direct relationship with the fragments which load the iron ions. In the early stage of the substrate molecular docking experiments, we found that the C-terminal helix of AnFNS I had a substrate-binding site (322 amino acids of AnFNS I). As such, we speculated that the C-terminal helix of FNS I had a certain influence on the conformation of the substrate in the active pocket. Lange et al. reported that the catalytic characteristics of 2OGDs could be significantly changed by adding GA20-oxidases to the C-terminus of 2OGDs (Lange et al., 1997). Therefore, mutant 03 was constructed to verify the influence of the C-terminal helix on the catalytic characteristics. It was found that at the C-terminal helix of AnFNS I, which was embedded in the C-terminus of CaFLS, no significant changes were observed between the ratio of diosmetin to hesperetin in the products (both around 5%). Notably, the overall catalytic activity of the substrate was reduced from 50.33 to 6.74%. Currently, there are contradicting findings on the effect of C-terminal helix truncation or extension. Chin et al. reported that the removal of 20 amino acids at the C-terminus of deacetylcephalosporin C synthetase did not significantly change the catalytic activity (Chin et al., 2003). However, Wellmann et al. indicated that the truncated C-terminal amino acids of Petunia hybrida FHT, did not significantly change the catalytic characteristics of FHT but reduced the total activity by 56–72% (Wellmann et al., 2002). Since the C-terminal helix is located near the entrance of the active pocket with the iron ligand as the center, it is considered that the C-terminal helix can determine whether the substrate can enter the active pocket. In addition, it can determine the spatial conformation of the substrate in the active pocket through its hydrophobicity, thus affecting the activity and catalytic characteristics of the enzyme.

### Influence of the Active Pocket

In this study, we compared the structure of FLS and FNS I from multiple sources and found active pockets, namely APD 2, that were more frequent with FNS I than FLS. APD 1 is the most reported catalytic pocket of 2-oxoglutarate-dependent dioxygenase (2OGDs) (Zeb et al., 2019). In addition, significant differences in the depth of the active pocket between AnFNS I and CaFLS were observed. The difference in depth may be caused by the amino acid residues at the tip of the active pocket and further affect the relative positioning of the loaded iron ions with the substrate. APD 2 is an active pocket commonly found in FNS I but not in CaFLS. Martens et al. predicted that FNS catalyzed the conversion of flavanones to flavones by the hydroxylation of C-3, followed by an elimination reaction resulting in flavones (Martens et al., 2003). Among the structural differences between AnFNS I and CaFLS at the entrance of APD 1, the electrostatic effects of few charged amino acid residues were different. These include AnFNS I-leu198 to CaFLS-lys198, AnFNS Ⅰ-Arg215 to CaFLS-Glu215, and AnFNS I-Arg128 to CaFLS-Ile126. The observed differences may influence the process by which the substrates enter APD 1. We then designed mutant 02 by replacing amino acids 236–241 of wild-type CaFLS to validate the function of specific active pockets of FNS I. This resulted in the formation of a similar active pocket to that of APD 2 in AnFNS I. However, mutant 02 did not have the ability to synthesize flavones in the bioconversion assay in *E. coli*. Although mutants 04 and 10 did not form an active pocket similar to APD 2, they showed better catalytic activity in the biosynthesis of diosmetin when compared with wild-type CaFLS. Therefore, APD 2 is nonessential for the enzyme to exert the ability to synthesize diosmetin. APD 1 which load iron ions is the key active pocket fo the synthesis of diosmetin. Since 2OGDs including FNS, FHT, and FLS are highly conserved at amino acids 217–296, we speculated that the active pocket with iron ligand as the center, formed by the two key conserved domains of DIOX_N and 2OG-Fe(II) oxygenase, are the key to understanding the enzyme activity. Moreover, the active pocket is solely responsible for the complete transformation of flavanone, flavanol, flavones, and flavonols.

### Influence of Relative Spatial Location

Mutants 04–10 were designed to study the effect of substrate-binding sites on the catalytic characteristics, whereas mutant 01 was designed to study the effect of active pocket depth on the catalytic characteristics. The results from the bioconversion assay in *E. coli* indicated that the distance from the carbonyl to H-2 was less than that to H-3, which was conducive to bioconversion at C-2 of hesperetin. The proportion of diosmetin or tamarixetin detected in the products was also increased in mutants 01 and 04. The distance from the carbonyl to H-2 was longer than that from the carbonyl to H-3, which was conducive to bioconversion at C-3 of hesperetin. As such, the catalytic activity observed in mutants 07 and 09 were similar to those of wild-type CaFLS. While the substrate could not penetrate the active pocket and the carbonyl was inaccessible to H-2/H-3, the catalytic activity of the mutant was significantly weakened in mutants 03 and 05. Therefore, the difference between the distance of the carbonyl and H-2/H-3 of hesperetin affected the catalytic characteristics, resulting in the transformation of flavanone to flavones as the predominant transformation. This is consistent with a previous study by Gebhart et al. (2007). However, in this study, it was found that reducing the distance from the carbonyl to H-2 in comparison with H-3 could not effectively influence the bioconversion of flavanol to flavones, since a large number of flavonols as by-products was not observed. The maximum proportion of diosmetin was obtained when the mutant could not effectively catalyze a reaction at C-3 because the flavonoid ring was between the carbonyl and H-3, as observed in AnFNS Ⅰ and mutant 10. In a report by Gebhardt et al. (2007), I131F was the key mutation in FNS activity. It was observed that the I131F point mutation did not change the catalytic characteristics of FHT, whereas in this study, the substitution of six amino acids near Y128 changed the catalytic characteristics of CaFLS to AnFNS I. Therefore, the change in the spatial conformation of the substrate in the active pocket caused by the change in the overall morphology of the active pocket may be the cause of the difference in catalytic characteristics. In addition, all mutants with differentially charged residues near the entrance of APD 1 (mutants 04, 08, and 10) exhibited highly significant changes in the proportion of diosmetin in the catalytic products. Significant increases in the proportion of diosmetin in the catalytic products of mutant 04 and 10 (from 2.53 to 10.62% and 16.68%, respectively). In contrast, mutant 08 completely lost its ability to synthesize diosmetin. Meanwhile, molecular docking results also revealed that the distance from H-2 to the carbonyl in the iron ligand was smaller than that from H-3 to the carbonyl in mutants 04 and 10. In mutant 08 however, the substrate was located on the other side of the flavonoid plane and displayed weakened H-2 catalytic activity.

This study provides evidence that the major differences in the catalytic characteristics of CaFLS and AnFNS I which as a metalloenzyme with loaded iron ions as the catalytic core depend on the spatial position of the substrate and the iron ligand in the active pocket. The relative position and distance of the carbonyl in the iron ligand to the H-2/H-3 of hesperetin determines whether the enzyme can catalyze transformations at the C-2/C-3 position of hesperetin, which determines the type of final product. This determinant is related to the charged residues at the entrance to the active pocket. It was observed that when the enzyme only had H-2 catalytic activity, the products were primarily flavonoids, as observed in mutant 10. Since H-2 has a stronger directionality than C-2, we consider that the atoms in the substrate molecule that determined the catalytic outcome are more likely H-2 and H-3, rather than C-2 and C-3 in as indicated in existing reports. As with H-2, when the enzyme only had H-3 catalytic activity, the products were primarily flavonoids, as observed in mutant 08. However, when the enzyme had simultaneous H-2/H-3 catalytic activity, as observed in mutant 04, flavonols were the primary product. Britsch et al. investigated the catalytic characteristics of FNS toward different chiral flavonoids and found that FNS could only catalyze the synthesis of apigenin from *2S*-naringenin but not *2R*-naringenin (Britsch 1990), due to the location of H-2. This supports our speculation that other than the distance, the spatial location of hesperetin and loaded carbonyl iron is more likely responsible for, or is the main factor for the differences in the catalytic characteristics of FNS and FLS. Based on these conclusions and conjectures, computational simulation may be used to assess and modify enzymes for future research. Saturation mutations of key amino acids and in silico techniques were used to analyze the effect of the mutations on the enzymes. This allowed for the rapid acquisition of more efficient mutants and reduced the amount of work required during enzyme evolution.

### Selection of Docking Modality and Model Reliability

It should be mentioned that when analyzing the substrate-binding sites in the different mutations, we used synchronous docking, iron ligand and hesperetin are used to dock with the protein respectively, rather than initially fixing the iron ligand on the enzyme structure and then docking with hesperetin. Multiple protein models with a sidechain bearing an iron ligand were constructed for step-by-step molecular docking in our initial study, and the docking results showed that hesperetin did not gain access to the active pocket APD 1. This did not correspond to the actual results and existing studies, since the binding of the protein to the ligand is not anchorage invariant. Therefore, we did not choose this docking method for the analysis. Although the protein models were not rigorously optimized after homology modeling, they were relatively stable and credible due to the high sequence consistency with model quality. Since the speculations made based on molecular docking matched the actual reaction results, we considered our hypothesis plausible.

## Data Availability

The original contributions presented in the study are included in the article/Supplementary Material, further inquiries can be directed to the corresponding authors.
