# Supplementary material for: Substrates and Loaded Iron Ions Relative Position Influence the Catalytic Characteristics of the Metalloenzymes Angelica archangelica Flavone Synthase I and Camellia sinensis Flavonol Synthase
Source: Front Pharmacol. 2022 Jun 8;13:902672. doi: 10.3389/fphar.2022.902672 (PMC9213739; doi:10.3389/fphar.2022.902672)
Supplement: Supplementary file 1 [file Table1.DOCX]

Fig. 1 ^1^H-NMR spectrum of diosmetin standard substance

Fig. 2 ^1^H-NMR spectrum of isolated diosmetin (from *E. Coli* carrying CaFLS)

Fig. 3 ^1^H-NMR spectrum of 4'-*O*-methyl taxifolin standard substance

Fig. 4 ^1^H-NMR spectrum of isolated 4'-*O*-methyl taxifolin (from *E. Coli* carrying CaFLS)

Fig. 5 ^1^H-NMR spectrum of tamarixetin standard substance

Fig. 6 ^1^H-NMR spectrum of isolated tamarixetin (from *E. Coli* carrying CaFLS)

Fig. 7 LC-MS spectrum of Hesperetin standard substance

Fig. 8 LC-MS spectrum of 4'-O-methyl taxifolin standard substance

Fig. 9 LC-MS spectrum of diosmetin standard substance

Fig. 10 LC-MS spectrum of tamarixetin standard substance

Fig. 11 LC-MS spectrum of negative control

Fig. 12 LC-MS spectrum of fermentation broth of recombinant *E. coli* carrying AnFNS Ⅰ
