# Supplementary material for: Substrates and Loaded Iron Ions Relative Position Influence the Catalytic Characteristics of the Metalloenzymes Angelica archangelica Flavone Synthase I and Camellia sinensis Flavonol Synthase
Source: Front Pharmacol. 2022 Jun 8;13:902672. doi: 10.3389/fphar.2022.902672 (PMC9213739; doi:10.3389/fphar.2022.902672)
Supplement: Supplementary file 2 [file Table2.DOCX]

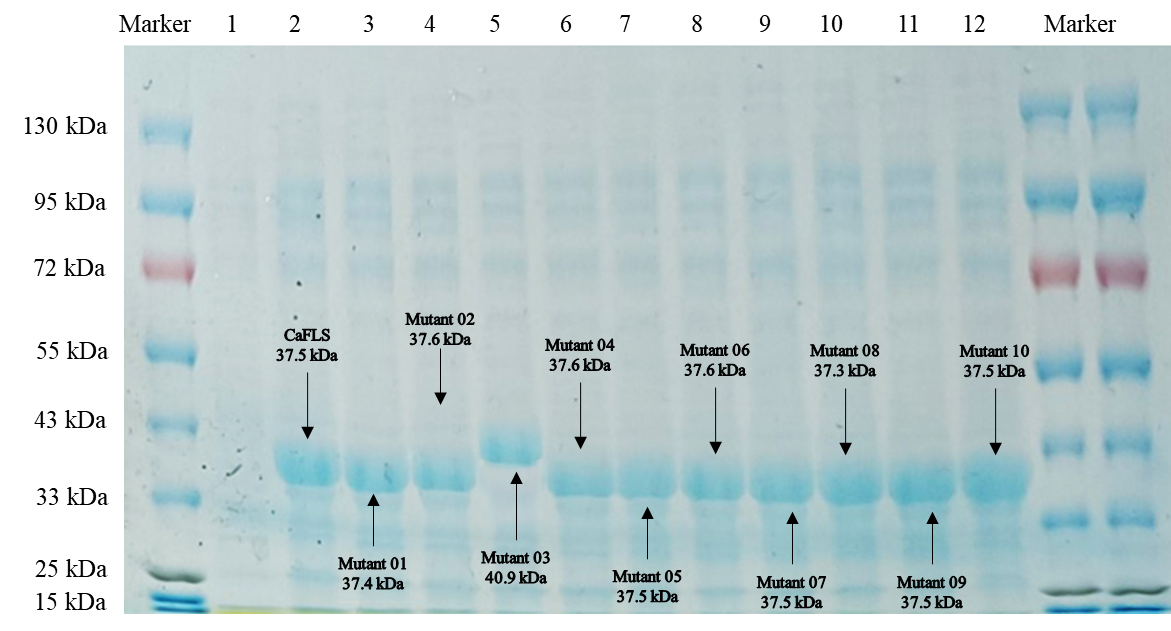


**Supplementary material 4 SDS-PAGE analysis of intracellular soluble proteins**

Note: **1** Recombinant *E. coli* not carring FLS gene, **2** Recombinant *E. coli* carrying the wild-type CaFLS gene, **3** Recombinant *E. coli* carrying the mutant 01 gene, **4** Recombinant *E. coli* carrying the mutant 02 gene, **5** Recombinant *E. coli* carrying the mutant 03 gene, **6** Recombinant *E. coli* carrying the mutant 04 gene, **7** Recombinant *E. coli* carrying the mutant 05 gene, **8** Recombinant *E. coli* carrying the mutant 06 gene, **9** Recombinant *E. coli* carrying the mutant 07 gene, **10** Recombinant *E. coli* carrying the mutant 08 gene, **11** Recombinant *E. coli* carrying the mutant 09 gene, **12** Recombinant *E. coli* carrying the mutant 10 gene.
